# Are aortic coarctation and rheumatoid arthritis different models of aortic stiffness? Data from an echocardiographic study

**DOI:** 10.1186/s12947-018-0126-y

**Published:** 2018-06-26

**Authors:** Giorgio Faganello, Giovanni Cioffi, Maurizio Rossini, Federica Ognibeni, Alessandro Giollo, Maurizio Fisicaro, Giulia Russo, Concetta Di Nora, Sara Doimo, Luigi Tarantini, Carmine Mazzone, Antonella Cherubini, Biancamaria D’Agata Mottolesi, Claudio Pandullo, Andrea Di Lenarda, Gianfranco Sinagra, Ombretta Viapiana

**Affiliations:** 1 Cardiovascular Centre, Department of Cardiology, Azienda Sanitaria Universitaria Integrata di Trieste, via Slataper n°9, 34134 Trieste, Italy; 2 Department of Cardiology, Villa Bianca Hospital, Trento, Italy; 3 Department of Medicine, Azienda Ospedaliera Universitaria Integrata di Verona, Verona, Italy; 4 Department of cardiology, Ospedale Civile S. Martino, Belluno, Italy; 5 Division of Cardiology, Institute for Maternal and Child Health, IRCCS Burlo Garofolo, Trieste, Italy; 6 Department of Cardiology, Azienda Sanitaria Universitaria Integrata di Trieste, Trieste, Italy

## Abstract

**Background:**

Patients who underwent a successful repair of the aortic coarctation (CoA) show high risk for cardiovascular (CV) events. Mechanical and structural abnormalities in the ascending aorta (Ao) might have a role in the prognosis of CoA patients. We analyzed the elastic properties of Ao measured as aortic stiffness index (AoSI) in CoA patients in the long-term period and we compared AoSI with a cohort of 38 patients with rheumatoid arthritis (RA) and 38 non-RA matched controls.

**Methods:**

Data from 19 CoA patients were analyzed 28 ± 13 years after surgery. Abnormally high AoSI was diagnosed if AoSI > 6.07% (95th percentile of the AoSI detected in our reference healthy population). AoSI was assessed at the level of the aortic root by two-dimensional guided M-mode evaluation.

**Results:**

CoA patients showed more than two-fold higher AoSI compared to RA and controls (9.8 ± 12.6 vs 4.8 ± 2.5% and 3.1 ± 2.0%, respectively; all *p* < 0.05 and in 5 of 19 patients with CoA (26%) AoSI was exceptionally high. The 5 patients with abnormally high AoSI were older with higher BP, LV mass and prevalence of LV diastolic dysfunction. Multiple linear regression analysis revealed that AoSI was independently related to the presence of LV hypertrophy and higher LV relative wall thickness.

**Conclusions:**

CoA patients have higher AoSI levels than RA patients and non-RA matched controls. AoSI levels are abnormally high in a small sub-group of CoA patients who show a very high-risk clinical profile for adverse CV events.

## Background

Despite successfully treated, patients with repaired aortic coarctation (CoA) have reduced long-term survival compared with an age and sex matched population [1]. The presence of high systemic blood pressure generally found in these patients together with altered integrity of the aorta due to surgical repair, and/or acquired post-surgery, cause progressive change in the structure and function of arterial vessels [2–4]. Therefore, it is common to find hypertrophy and hyperplasia of smooth muscle cells, together with modification of the matrix protein [5]. The continuous deposition of a variety of proteins, including collagen, combined with the progressive loss of the elastic matrix leads to a further increase in arterial stiffness and reduction of vascular compliance [6]. Similar features have been found in patients affected by rheumatoid arthritis (RA) which represents a clinical model of acquired extensive arterial disease with abnormal vascular responses [7] to inflammation and/or damaging molecular modulators of immune system. Abnormal aortic elastic properties are associated with older age and higher blood pressure in RA patients [8]. The result of these changes is stiffening of the arteries and consequent increase of aortic stiffness index (AoSI) which is used to evaluate arterial stiffness and it is an independent predictor of cardiovascular morbidity and mortality [9]. AoSI showed a strong correlation with the invasive measurements of arterial stiffness and more popular non-invasive techniques such as pulse wave velocity or Tissue Doppler [10–13]. Accordingly, in this study we measured the AoSI of the ascending aorta in patients with successful CoA repair in the long-term period and we compared it with a cohort of RA patients.

## Methods

### Study population

The study population consisted of 19 non-institutionalized subjects > 18 years of age who consecutively underwent successful repair of CoA during the period from 1964 to 2010 and were subsequently followed-up by the Institute for Maternal and Child Health-IRCCS, Burlo Garofolo and at the Cardiovascular (CV) Center, Maggiore Hospital, Trieste, Italy. Repair of CoA was obtained by: end-to end anastomosis in 9 patients, patch in 4 patients, subclavian flap in 2 patients, subclavian artery flap in 1 patient and percutaneous stent in 3 patients. The mean duration of follow-up was 28 ± 13 years. The patients’ inclusion criteria were:

- Residual isthmic gradient by echo-Doppler less than 20 mm Hg;
- Aortic diameter at the site of repair/diaphragmatic aorta ratio more than 0.7 at cardiac MRI
- No associated cardiac abnormalities or moderate/severe valve heart disease;
- No bicuspid aortic valve with moderate/severe regurgitation or stenosis;
- Absence of history of myocardial infarction or prior myocardial revascularization, asymptomatic known LV dysfunction, heart failure, primary cardiomyopathies or myocarditis, atrial fibrillation, chronic kidney disease, obstructive sleep apnea syndrome, RA (all conditions eliciting changes in LV geometry and LV systolic dysfunction). All patients underwent transthoracic echocardiogram, clinical and laboratory evaluation at the CV Center, Maggiore Hospital, Trieste, Italy. Patients expressed their general written consent to the anonymous use of data for their care and research purposes. The study complies with the Declaration of Helsinki as revised in 2000; the locally appointed ethic committee has approved the research protocol.

### Rheumatoid arthritis group

A group of 38 patients matched for age, sex, blood pressure, history of hypertension and affected by RA was identified. RA was diagnosed by clinical and laboratory examination according to the American College of Rheumatology criteria [14]. They were selected by a large cohort of patients consecutively recruited from January 2014 to December 2014 in three Italian referral centers (Verona, Trieste, Trento) with fully accessible cardiac units provided in which patients underwent echocardiographic, clinical and laboratory evaluations.

### Control group

A control group of 38 subject defined “non-RA patients” matched for age, sex, blood pressure and history of hypertension was identified. RA patients and non-RA controls were studied for the reason to assess the range of values of aortic stiffness in a model of abnormal vascular responses and of acquired chronic arterial disease, respectively. Controls were free of symptoms/signs of cardiac disease and had no history of myocardial illness or valve heart disease, including evidence of more than mild mitral annular calcification at echocardiographic baseline evaluation. These two groups of subjects were statistically comparable with those enrolled into the study for age, sex, blood pressure and hypertension according to the following procedure: a Gower’s generalized distance from each of the RA individual and each CoA patient was computed and all patients were ranked in ascending order in the database. The distance was calculated using these variables ordered as follows: age, gender, systolic blood pressure and hypertension. The 38 RA patients and 19 CoA patients were then demarcated and coupled by taking for everyone patient with CoA the two closest controls (selected by a pool of 250 patients). Then, the 19 patients with CoA were compared with a second control group (defined non-RA matched controls), composed of 38 patients, matched for age, sex, blood pressure and prevalence of hypertension by the same statistical procedure described above. The 38 non-RA matched controls were designated by taking for everyone close patient with CoA the two closest control. These subjects were selected by a pool of 180 patients who consecutively performed at our Center a clinical and an echocardiographic evaluation for a CV risk assessment in primary prevention.

### Definitions

Arterial hypertension was defined as systolic blood pressure of ≥140 mmHg and/or a diastolic blood pressure of ≥90 mmHg and/or pharmacologically treated high blood pressure. Obesity was diagnosed if patients had body mass index ≥30 kg/m2. Dyslipidemia was defined as levels of total serum cholesterol > 190 mg/dl and or triglycerides > 150 mg/dl or pharmacologically treated high lipid serum levels.

### Echocardiography

LV chamber dimensions and wall thicknesses were measured by the American Society of Echocardiography guidelines and LV mass calculated using a necropsy validated formula [15]. LV mass was normalized for height to the 2.7 power and LV hypertrophy was defined as LV mass > 49.2 g/m2.7 for men and > 46.7 for women [16]. Relative wall thickness was calculated as the 2 * end-diastolic ratio posterior wall thickness/LV diameter and indicated concentric LV geometry if > 0.43 (the 97.5 percentile in normal population) [17] LV end-diastolic and end-systolic volumes were measured by the biplane method of disks from 2D apical 4 chamber + 2 chamber views and used to calculate ejection fraction, defined as index of global LV systolic function measured at endocardium. LV ejection fraction < 50% was indicative of LV systolic dysfunction (LVSD). LV systolic function was also assessed by measuring the systolic shortening of the LV minor axis at the midwall level to specifically evaluate the circumferential component of LV systolic function. Midwall shortening was calculated considering the epicardial migration of the midwall during systole caused by the architectural organization of myocardial fibers, as previously described [18]. Midwall end-systolic circumferential stress (sc-MS) was calculated and related to midwall shortening to assess afterload-independent LV systolic function [19]. Thus, sc-MS refers to the ratio observed/predicted midwall shortening for a given end-systolic circumferential stress and corrects for this variable the LV systolic function. sc-MS < 89% (10th percentile of our healthy controls) was indicative of circumferential LVSD. Furthermore, Tissue Doppler study (pulsed wave spectral analysis) was used to measure peak mitral annular systolic velocity (peak S’, mean of 4 measurements obtained in septal, lateral, inferior and anterior mitral annular position), as an estimate of longitudinal component of LV systolic function [20]. Peak S’ < 8.5 cm/sec (10th percentile of our healthy controls) indicated longitudinal LVSD. Transmitral and pulmonary vein pulsed wave Doppler curves and early diastolic Tissue Doppler velocity of mitral annulus (E’) were assessed according to the recommendations of the American Society of Echocardiography [21]. Early diastolic velocity of transmitral flow (E) was divided by E’ and used to classify LV diastolic function together with other parameters (E/A ratio of transmitral flow, deceleration time of E and the difference in duration of atrial wave on pulmonary vein flow and atrial wave on transmitral flow) in 4 degrees as proposed by Redfield et al. [22]: normal, mild dysfunction, moderate dysfunction and severe dysfunction. LV end-diastolic pressure was non-invasively estimated by the equation validated by Ommen et al. [23]. Maximal left atrial volume was also computed from 2D apical 4-chamber view using the area - length method and was normalized for body surface area. The residual isthmic gradient was assessed with a standalone, 2.0-MHz, continuous-wave Doppler probe using the modified Bernoulli equation: gradient (mmHg) = 4(V_2_^2^ - V_1_^2^) m/s, where V_2_ was the maximum velocity in the descending aorta and V_1_ was the velocity in the descending aorta above the coarctation site when a double shadow could be obtained on Doppler tracing, or V1 was the velocity in the ascending aorta when a suitable tracing was not obtained [24]. Doppler gradients were considered as the mean of at least 3 consecutive measurements.

### Calculation of aortic stiffness

Aortic stiffness was assessed at the level of the aortic root, using a two-dimensional guided M-mode evaluation of systolic (AoS) and diastolic (AoD) aortic diameters, 3 cm above the aortic valve together with blood pressure measured by cuff sphygmomanometer. AoD was obtained at the peak of the R wave at the simultaneously recorded electrocardiogram, while AoS was measured at the maximal anterior motion of the aortic wall [25]. For each diameter five measurements were averaged. The following formula was used for assessing aortic stiffness index (AoSI):

$$ \left(\mathrm{AoS}\mathrm{I}\right)\ \left(\%\right)\kern0.75em =\kern0.75em \ln \left(\mathrm{SBP}/\mathrm{DBP}\right)/\left[\left(\mathrm{AoS}-\mathrm{AoD}\right)/\mathrm{AoD}\right] $$

where ln[systolic blood pressure (SBP)/diastolic blood pressure (DBP)] refers to the natural logarithm of the relative blood pressure (SBP and DBP: systolic and diastolic blood pressure) [26]. Blood pressure was measured at the end of echocardiographic evaluation in supine position. AoD and AoS were evaluated off-line by the principal investigator blinded to the identity of the subject. Reproducibility data on AoSI assessment have been previously reported [27].

### Cardiac magnetic resonance

The degree of residual coarctation was determined by spin-echo magnetic resonance imaging of the thoracic aorta. The smallest diameter was measured by hand calipers from internal edge to internal edge of the vascular walls from a combination of 2 views: transverse and sagittal oblique (left anterior oblique equivalent) through the center of the vessel. The smallest diameter was compared with the diameter of the aorta at the diaphragm. Percent narrowing was calculated as: % narrowing = 100 (1 - smallest diameter/diameter at diaphragmatic level).

### Statistical analysis

Categorical variables are presented as percentages, while continuous variables are presented as their means and SD. Categorical variables were compared by the chi-square test and continuous variables by the t-test or the Mann–Whitney U-test. The study population was also stratified by status of abnormally high AoSI at baseline. The cut-off value for abnormally high AoSI was a priori identified as 6.07% (the 95th percentile of AoSI calculated in the 113 healthy subjects) as previously reported. Multiple linear regression analysis was computed to assess the variables significantly related to AoSI index in CoA patients. Variables significantly related to AoSI in univariate tests (*p* < 0.01) were considered in the multivariable model, which included age, body mass index, systolic blood pressure, E/E’, LV relative wall thickness and LV hypertrophy. All analyses were performed using statistical package SPSS 19.0 (SPSS Inc. Chicago. Illinois) and statistical significance was identified by two-tailed *p* < 0.05.

## Results

### Study population

All subjects were free of symptoms and clinical signs of cardiac disease at the time of clinical, laboratory and echocardiographic evaluation. During the 28 ± 13 years of follow-up after surgical intervention, no patient who had undergone successful repair of CoA suffered from an adverse CV event either was hospitalized for any sign or symptoms potentially related to the presence of CV disease. Similarly, RA and non-RA matched controls were analyzed in primary prevention. Clinical and echocardiographic characteristics of the study population compared with RA patients (mean time from the RA diagnosis 14 ± 10 years) and non-RA matched controls are shown in Tables 1 and 2, respectively. Between all groups, there were no statistical differences in terms of age, sex, body mass index, laboratory data and prevalence of dyslipidemia, type 2 diabetes mellitus and blood hypertension. Despite RA subjects were taking less anti-hypertensive medications compared to CoA patients and controls, blood pressure values were similar between the groups. All groups had LV dimensions within the normal range, however CoA patients showed higher prevalence of LV hypertrophy than controls and RA patients (37% vs 20% vs. 6%, respectively; all *p* < 0.05) associated with a higher end-systolic circumferential stress and lower relative wall thickness, index of concentric LV geometry. Regarding to the parameters of LV systolic function, all three groups showed similar LV ejection fraction and sc-MS (parameter of LV circumferential systolic function), however peak S’ (parameter of longitudinal LV systolic function), was significantly lower in CoA subjects than controls and RA patients (7.1 ± 1.3 vs. 10.5 ± 3.6 vs. 10.2 ± 1.6 cm/sec; *p* < 0.05). CoA patients had a greater prevalence of LV diastolic dysfunction as shown by raised E/E’ ratio and end-diastolic pressure.

**Table 1:** Main clinical characteristics of the 19 study patients with aortic coarctation compared with 38 controls matched for cardiovascular risk factors and 38 patients with rheumatoid arthritis

Variables	Aortic coarctation (19 patients)	Controls (38 patients)	RA (38 patients)
Clinical			
Age (years)	33 ± 12	35 ± 14	37 ± 6
Female gender (%)	37	32	48
Body mass index (Kg/m^2^)	23.9 ± 4.0	26.8 ± 4.4	24.2 ± 4.2
Obesity (%)	11	17	9
Hypertension (%)	58	65	41
Dyslipidemia (%)	5	5	15
Active smoker (%)	21	12	28
Diabetes (%)	5	5	3
Systolic blood pressure (mmHg)	127 ± 17	131 ± 19	125 ± 16
Diastolic blood pressure (mmHg)	77 ± 9	83 ± 10	82 ± 10
Heart rate (beats/minute)	68 ± 12	71 ± 14	76 ± 10
Laboratory			
Glycemia (mg/dl)	93 ± 9	98 ± 10	93 ± 9
Hemoglobin (gr/dl)	13.8 ± 1.6	14.6 ± 1.7	14.0 ± 1.2
GFR (ml/min/1.73m^2^)	105 ± 26	103 ± 20	108 ± 26
Pharmacological treatment			
Betablockers (%)	32	22	8 ^**# §**^
ACEi / ARB (%)	32	52	12 ^**# §**^
Diuretics (%)	16	30	3 ^**# §**^
Calcium antagonists (%)	11	35	3 ^**# §**^
Anti-platelets agents (%)	0	1	4
Statins (%)	0	1	1

*ACEi* Angiotensin-converting enzyme inhibitors, *ARB* Angiotensin T1 receptor blockers, *GFR* Glomerular Filtration Rate*, RA* rheumatoir arthritis

^#^ = *p* < 0.05 rheumatoid arthritis (RA) vs coarctation

^§^ = *p* < 0.05 RA vs controls

**Table 2:** Echocardiographic characteristics

Variables	Aortic coarctation (19 patients)	Controls (38 patients)	RA (38 patients)
LV End-diastolic diameter (ml/m^2^)	2.9 ± 0.3	2.3 ± 0.4 *	2.6 ± 0.3^**#**^
LV End-systolic diameter (ml/m ^2^)	1.9 ± 0.3	1.4 ± 0.4 *	1.7 ± 0.2^**#**^
LV End-diastolic volume (ml/m^2^)	66 ± 19	56 ± 12	51 ± 9 ^**# §**^
LV End-systolic volume (ml/m^2^)	27 ± 9	21 ± 6	17 ± 4 ^**# §**^
Relative wall thickness	0.35 ± 0.06	0.40 ± 0.07 *	0.43 ± 0.06 ^**# §**^
Concentric LV geometry (%)	11	45 *	51 ^**#**^
LV mass index (g/m ^2.7^)	41 ± 14	38 ± 13	38 ± 7^**#**^
LV hypertrophy (%)	37	20 *	6 ^**# §**^
Inappropriate LV mass (%)	11	10	36 ^**# §**^
LV stroke volume (ml)	73 ± 25	74 ± 18	59 ± 15 ^**# §**^
Cardiac index (l/min/ m^2^)	2.7 ± 0.9	2.4 ± 0.7	2.3 ± 0.6^**#**^
LV ejection fraction (%)	60 ± 7	63 ± 5	65 ± 5^**#**^
LV CESS (dynes/cm^2^)	166 ± 49	128 ± 48 *	116 ± 28^**#**^
LV Sc- midwall shortening (%)	101 ± 15	98 ± 10	85 ± 11
Peak S’ (cm/sec)	7.1 ± 1.3	10.5 ± 3.6 *	10.2 ± 1.6^**#**^
Low peak S’ (%)	84	55 *	27^**# §**^
Peak E’ (cm/sec)	10.6 ± 3.5	12.3 ± 1.7	13.6 ± 2.2
E wave of transmitral flow (cm/sec)	103 ± 16	86 ± 19 *	75 ± 17^**# §**^
A wave of transmitral flow (cm/sec)	69 ± 25	64 ± 16	62 ± 15
E / A ratio	1.4 ± 0.36	1.4 ± 0.24	1.3 ± 0.40^**#**^
E / E’ ratio	10.5 ± 3.6	7.5 ± 2.3 *	5.6 ± 1.1^**# §**^
LV diastolic dysfunction (%)	11	2 *	3^**#**^
Maximal left atrial volume (ml/ m^2^)	27 ± 11	23 ± 11	18 ± 4
Aortic stiffness index (%)	9.8 ± 12.6	3.1 ± 2.0 *	4.8 ± 2.5^**#**^
Abnormally high aortic stiffness (% of patients)	26	10	21

*CESS* circumferential end-systolic stress, *LV* left ventricular; *Peak E’*early diastolic Tissue Doppler velocity of mitral annulus, *Peak S’* peak mitral annular systolic velocity (Tissue Doppler Imaging), *RA* rheumatoid arthritis, *Sc* stress corrected

^*^ = *p* < 0.05 controls vs coarctation;

^#^= *p* < 0.05 rheumatoid arthritis vs coarctation;

^§^ = *p* < 0.05 rheumatoid arthritis vs controls

### Aortic arterial stiffness

**Fig. 1:**
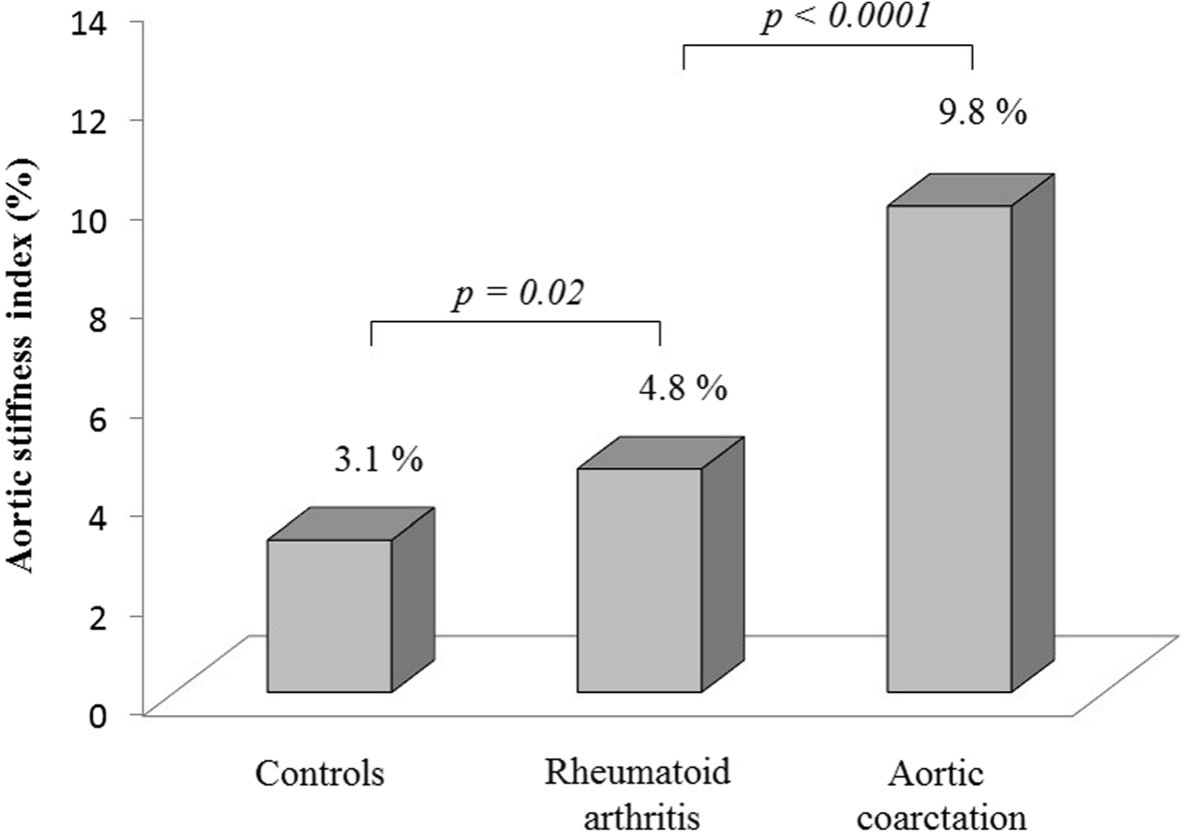
Comparison of AoSI between CoA group, RA subjects and controls

**Fig. 2:**
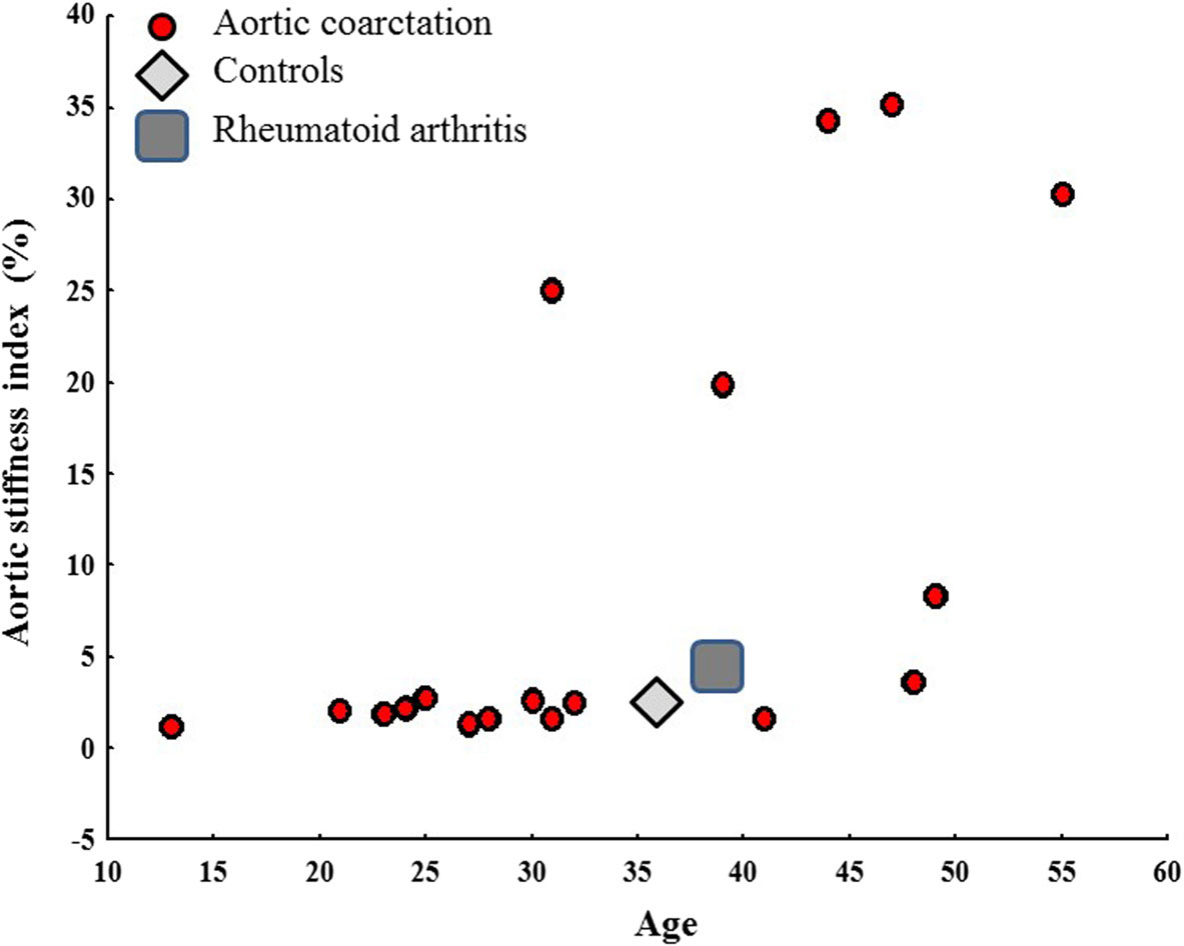
Distribution of AoSI between all groups. 5 CoA patients have exceptionally high AoSI

AoSI was significantly higher in the CoA group compared to RA subjects (9.8 ± 12.6% vs. 4.8 ± 2.5%, *p* < 0.0001) and in turn, RA subjects had increased values compared to non-RA matched controls (4.8 ± 2.5% vs. 3.1 ± 2.0, *p* = 0.02) (Fig. 1). The marked increase in AoSI found in CoA patients was essentially due to the presence of 5 subjects showing abnormally high AoSI (mean value 28.9 ± 6.5%) in comparison of the remaining 14 who had AoSI values in the normal range (2.5 ± 1.9%) (Fig. 2). The clinical and echocardiographic characteristics of CoA patients with and without abnormally high AoSI are shown in Table 3. Among CoA group, patients who had abnormally high AoSI were older, with higher blood pressure values, body mass index, LV mass and worse diastolic function. Four out of five patients were treated with end-to-end anastomosis and only in one case a dacron-patch was used. Multiple linear regression analysis revealed that AoSI was independently related to LV hypertrophy and higher LV relative wall thickness, index of concentric LV geometry (Table 4). Considering the control group, abnormally high AoSI was detected in 4 of 38 (10%) and in 5 of 38 patients with RA (21%). Among the three groups there were no statistically significant differences in the size of the aortic root.

**Table 3:** Variables significantly different between aortic coartaction patients who had abnormally high aortic stiffness and those who had not

Total study population (19 patients)	Abnormally high aortic stiffness NO (14 patients)	Abnormally high aortic stiffness YES (5 patients)	*p*
Age (years)	30 ± 10	43 ± 9	0.02
Body mass index (Kg/m^2^)	22.2 ± 2.7	27.7 ± 4.5	0.004
Systolic blood pressure (mmHg)	120 ± 14	144 ± 14	0.004
Diastolic blood pressure (mmHg)	73 ± 9	83 ± 10	0.04
E / E’ ratio	9.5 ± 2.1	13.7 ± 5.1	0.02
LV end-diastolic pressure (mmHg)	14 ± 3	19 ± 6	0.02
Relative wall thickness	0.33 ± 0.04	0.41 ± 0.04	0.002
LV mass index (g/m ^2.7^)	36 ± 13	56 ± 5	0.006
LV hypertrophy (%)	15	100	< 0.001
Aortic stiffness index (%)	2.5 ± 1.9	28.9 ± 6.5	< 0.001

E/E’ ratio = ratio between peak of early (E) wave of transmitral flow and peak (E’) early diastolic Tissue Doppler velocity of mitral annulus

**Table 4:** Variables significantly related to aortic stiffness index (expressed as continuous variable): multiple linear regression analysis

	Standardized coefficients beta	*P*
Left ventricular hypertrophy	0.62	< 0.001
Left ventricular relative wall thickness	0.34	0.04
Final results multivariate regression model Intercept = − 25.0 Standard error of estimation = 6.9 r ^2^ = 0.74	0.86	< 0.001

## Discussion

In our study, we analyzed AoSI after three decades of follow up in patients who underwent successful CoA repair and we compared it with two different cohorts of patients: the first one, non-RA patients matched for age, sex, blood pressure and history of hypertension, and the other one, affected by RA. Three main and original findings emerged by our analyses: 1) AoSI was significantly higher in CoA patients than in RA patients or non-RA matched patients; 2) increased AoSI was not homogeneous in CoA patients: two distinct groups, indeed, were identified, the first including near a quarter of subjects who had abnormally high values of AoSI, the second including the remaining three quarter of subjects who had values of AoSI in the normal range; 3) in CoA patients, AoSI was independently related to LV hypertrophy and concentric LV geometry.

We previously demonstrated persistence of reduced systolic LV long axis and diastolic functions in the long run after successful repair of CoA. In addition, Lam et al. showed that systolic LV long axis dysfunction was associated with increased AoSI in adult patients with corrected CoA, independently from other potential confounders such as hypertension and associated bicuspid aortic valve. More recently, Voges et al. demonstrated a combination between the impairment of elastic properties in the thoracic aorta with the remodeling of the common carotid artery in young patients nearly fifteen years after CoA repair [28]. Collectively, these findings suggest that CoA might determine aortic wall changes as a systemic vessel disease in humans. Using a clinically representative rabbit model of CoA and correction, Menon et al. [29] quantified mechanical alterations from a 20-mmHg blood pressure gradient in the thoracic aorta and related the expression of key smooth muscle contractile and focal adhesion proteins with aortic remodeling, reduced relaxation and increased stiffness. These structural and functional changes were attributed to a significant increase in non-muscle myosin and reduced smooth muscle myosin heavy chain expression in the proximal arteries of CoA which was not reversed upon blood pressure correction. Furthermore, Gardiner et al. [30] found that, arterial dilation induced by glyceryltrinitrate was significantly impaired in the pre-coarctation vascular bed of young adults who had undergone successful repair of coarctation in childhood. These results may be an important contributor to rest and/or exercise-related hypertension and late morbidity or mortality of these subjects.

Arterial stiffness and AoSI are independent predictors of cardiovascular morbidity and mortality [9]. A number of previous studies confirmed that non-invasive AoSI had a strong correlation with invasive measurements of arterial stiffness, and that the aortic elastic properties deteriorate in patients with coronary artery disease [10, 12]. Vitarelli et al. showed that the measurement of AoSI allows to differentiate hypertensive from healthy adults [11]. In addition, AoSI is increased in women with a previous pregnancy complicated by early onset of pre-eclampsia. Recently, Said et al. demonstrated that AoSI measured by photofletmismography is an independent predictor of cardiovascular events and mortality in a UK large community-based population [9]. From the analysis of our AoSI data, two different phenotypes of repaired CoA patients emerged, corresponding to two distinct pathophysiological models. CoA patients with abnormally high AoSI, indeed, were largely different in comparison with the counterparts with normal AoSI, being older, with higher body mass index, blood pressure, LV mass remodeled in a concentric fashion, and with worse LV diastolic function. In these patients, greater LV hypertrophy and relative wall thickness were possibly due to the persistent LV pressure-overload. So, an older age at the time of intervention seems to promote the development of sky high AoSI more than nearly three decades of follow up with well controlled blood pressure. In our study, we compared LV properties and AoSI in CoA and RA patients. Both diseases are characterized by a lower life expectancy owing to premature CV diseases. RA is a model of acquired extensive arterial disease with abnormal vascular responses due to a number of cytotoxic agents, pro-inflammatory and immuno-modulatory molecules and hyper-functioning neuro-hormonal systems. In line with previous experiences, RA patients had a prevalence of abnormally high AoSI nearly two-fold higher than controls matched for the traditional CV risk factors. Although an increased AoSI is a strong predictor of adverse clinical outcome at mid-term follow-up, our RA subjects were less treated with medications compared to CoA and controls. This data is coherent with that derived by the EPIDAURO registry showing the CV risk stratification missing in daily clinical practice of RA patients [31]. CoA and RA represent different pathophysiological models of aortic disease. The first one is an acquired structural damage primarily caused by hemodynamic factors of longstanding distal obstruction that lead to higher collagen load with a lower elastin and smooth muscle content [32, 33]. The second one is related to non-hemodynamic factors such pro-inflammatory, immunomodulatory, and cytotoxic agents that accelerate vascular atherosclerosis, myocardial fibrosis, and apoptosis by way of direct damage of the myocardial tissue. Despite these different models and magnitude of AoSI, we found several common points. The reduced long-term survival unites CoA and RA patients in the follow up. Persistence or newly developed systemic hypertension and AoSI may cause significant abnormalities in the CV system of both diseases such as LV remodelling and dysfunction, thereby compromising the prognosis.

### Study limitations

This study has several limitations. Firstly, it was not adequately powered to determine the effect of other potential confounders (clinical significance of very mild residual narrowing at CoA site, difference between surgical or endovascular treatment approach and usage of antihypertensive medications) on central aortic elastic properties. Secondly, due to the small sample size of the study population, we cannot draw any prognostic inference regarding the detection of abnormally high AoSI condition. Furthermore, the evaluation of AoSI by central pulse pressure as assessed by radial artery tonometry and pulse wave analysis instead of the brachial artery pulse pressure might be more accurate than M-mode-derived aortic recordings and brachial blood pressure we used. Strengths of our study include the very long-term follow-up, the consecutive enrollment of patients, the reliable and appropriate method utilized for the assessment of AoSI, and the comprehensive nature of the dataset.

## Conclusions

CoA patients have higher AoSI levels than RA patients and non-RA matched controls. AoSI levels are abnormally high in a small sub-group of CoA patients who show a very high-risk clinical profile for adverse CV events. Although CoA and RA represent two different pathophysiological models of CV disease, they are characterized by common detrimental consequences on LV function and aortic properties. In CoA patients, intervention at an early age before potentially irreversible structural changes to proximal aorta, might be clinically relevant while in RA patients, appropriate medical treatment to reduce AoSI, might preserve LV geometry and function. These can only be speculative conclusions: nonetheless, the results of this study reinforce the importance of the notion that the measurement of AoSI in CoA and RA patients should conceivably be introduced into routine clinical practice.

## Abbreviations

AoD: Diastolic aortic diameter
AoS: Systolic aortic diameter
AoSI: Aortic stiffness index
CoA: Aortic coarctation
CV: cardiovascular
DBP: Diastolic blood pressure
E: Early diastolic velocity of transmitral flow
E’: Early diastolic Tissue Doppler velocity of mitral annulus
LV: Left ventricular
LVSD: LV systolic dysfunction
RA: Rheumatoid arthritis
S′: Mitral annular systolic velocity
SBP: Systolic blood pressure
sc-MS: Midwall end-systolic circumferential stress
